# ACLD patients exhibit additional knee kinematic asymmetries at the speed level of healthy subjects

**DOI:** 10.3389/fbioe.2022.930722

**Published:** 2022-08-23

**Authors:** Lingchuang Kong, Tao Yang, Qing Wang, Yongliang Ou, Huayang Huang, Wenhan Huang, Tao Zhang, Yu Zhang, Xiaolong Zeng

**Affiliations:** ^1^ Department of Orthopaedics, General Hospital of Southern Theater Command, Guangzhou, China; ^2^ Department of Orthopaedics, Guangdong Provincial People’s Hospital, Guangdong Academy of Medical Sciences, Guangzhou, China; ^3^ Department of Orthopaedic Surgery, The Second Affiliated Hospital, Guangzhou Medical University, Guangzhou, China; ^4^ School of Medicine, South China University of Technology, Guangzhou, China

**Keywords:** anterior cruciate ligament deficiency, non-operative treatment, walking speed, kinematic asymmetries, knee

## Abstract

Anterior cruciate ligament deficiency (ACLD) patients tend to walk slowly but try to catch up with the speed level of healthy subjects daily. Exploring the effects of the walking speed level of healthy subjects on the ACLD patients’ knee kinematics is important to improving non-operative treatments and delaying the progression of posttraumatic knee osteoarthritis. This study aimed to explore whether healthy controls’ walking speed level leads to additional knee kinematic asymmetries in patients with ACLD. 27 ACLD patients and 29 healthy controls were recruited for the study. The ACLD patients walked at two levels of walking speed, including self-selected and healthy controls’ walking speed levels. A three-dimensional gait analysis system was used to collect their knee kinematic data. ACLD patients exhibited more kinematic asymmetries when walking at healthy controls’ walking speed level than at their self-selected speeds. The kinematic asymmetries included increased posterior tibial translation (4.6 mm) and anteroposterior tibial ROM (3.9 mm), abduction angle (1.5°), and distal tibial translation (3.2 mm) asymmetries (*p* < 0.05). Our findings are meaningful for developing non-operative treatment strategies for patients with ACLD. To get fewer knee kinematic asymmetries, self-selected walking speed could be suggested for patients with ACLD daily rather than the speed levels of healthy subjects.

## Introduction

The anterior cruciate ligament (ACL) is one of the dominant structures stabilizing the native knee joint. Over the past decades, the prevalence of anterior cruciate ligament deficiency (ACLD) has been increasing (Herzog et al., 2018). In addition to ACL reconstruction, nonoperative treatments are acceptable in patients with ACLD (Grindem et al., 2012; Grindem et al., 2014; Diermeier et al., 2021). A randomized controlled trial by Frobell et al. showed no significant clinical differences in both nonoperative and operative treatments in active young patients with isolated ACLD concerning patient-reported outcomes in the follow-up time of up to 5 years (Frobell et al., 2010; Frobell et al., 2013). Nevertheless, abnormal knee mechanics and long-term health problems (e.g., posttraumatic osteoarthritis and PTOA) remain concerns among ACLD patients. Knee kinematic asymmetries during walking are important mechanical alterations in ACLD patients and have been explored by researchers for decades (Von Porat et al., 2007; Tashman et al., 2008; Scanlan et al., 2010; Zampeli et al., 2018; Agostinone et al., 2021; Zampeli et al., 2021). Furthermore, knee kinematic asymmetries have been widely reported to be correlated with the development of PTOA (Stergiou et al., 2007; Andriacchi et al., 2009; Haughom et al., 2012; Li et al., 2018). Scholars have formed evidence-based theories that kinematic alterations during gait may play an important role in the onset of PTOA (Stergiou et al., 2007; Andriacchi et al., 2009).

Walking speed is a daily, basic, and relevant living issue for patients. Patients with knee diseases tend to walk more slowly than healthy subjects due to their functional limitations and protective strategies for knee health (Gibbs et al., 1993; Fransen et al., 1997; Stergiou et al., 2004; Zeni and Higginson, 2009; Huang et al., 2017). Zeni and Higginson suggested that decreasing their walking speeds may be an effective strategy for ACLR patients to relieve gait asymmetries and promote knee cartilage health (Zeni and Higginson, 2009). However, most ACLD patients are young and active people who often have busy work lives and fast-paced lives. For example, they might match the walking speeds of their healthy peers. As a result, they often struggle when they walk faster than their self-selected comfortable speed. Walking speed is reported to significantly affect knee walking kinematics (Hanlon and Anderson, 2006; Chehab et al., 2017). The knee kinematics of ACLD patients may also be significantly influenced by walking speed. Knee kinematic alterations may lead to the pathological progression of osteoarthrosis (Stergiou et al., 2007; Andriacchi et al., 2009). The knee kinematic changes induced by gait speed may become a concerning issue among ACLD patients.

Some researchers have explored the effects of walking speed on the gait asymmetries of ACL-deficient subjects (Tibone et al., 1986; Stergiou et al., 2004; Fuentes et al., 2011; Yim et al., 2015; Nazary-Moghadam et al., 2019; Stoelben et al., 2019; Garcia et al., 2021). Nazary-Moghadam et al. found that fast walking speeds can significantly decrease flexion-extension Lyapunov Exponent (a functional stability parameter) in ACLD patients compared to healthy controls (Nazary-Moghadam et al., 2019). They suggested walking fast can be challenging for patients with ACLD (Nazary-Moghadam et al., 2019). Fuentes et al. found that ACLD knees could not exhibit pivot-shift avoidance gait to prevent anterolateral rotatory knee instability during the terminal stance phase while walking fast. They suggested that ACLD patients cannot adapt their knee biomechanics as effectively during fast walking as healthy controls (Fuentes et al., 2011). However, there is limited data on whether fast walking can increase kinematic disorders in ACLD patients. Walking fast is highly demanding concerning knee function. It was reported that walking as fast as healthy controls may reveal gait asymmetries and functional disorders among patients with ACLD (Tibone et al., 1986; Fuentes et al., 2011; Garcia et al., 2021). It is also possible that additional knee kinematic asymmetries could be revealed among ACLD patients when they are walking fast. This could enhance the understanding of the biomechanics of ACLD knees and provide new information for non-operative ACLD therapy.

Above all, it remains unclear whether walking at the speed level of healthy individuals could contribute to knee kinematic asymmetries in patients with ACLD. Level walking is the most frequent and relevant functional activity. The study explored whether walking speed levels of healthy individuals significantly would increase knee kinematic asymmetries of ACLD patients. ACLD patients are characterized by anteroposterior disorders. We hypothesized that the walking speed level of healthy subjects could increase anteroposterior knee kinematic asymmetries.

## Methods

### Subjects

The study was approved by the ethics committee of the hospital. Informed consent was obtained from each participant. The inclusion criteria for patients were the following: (1) the presence of unilateral, isolated ACLD confirmed *via* magnetic resonance imaging, (2) no history of multi-ligament or meniscus injuries, and (3) no radiographic sign of PTOA. Patients were excluded if they had (1) musculoskeletal or neurological diseases that affect the lower limbs or (2) surgery or severe lower limb injury or deformity before/after ACL injury. Healthy controls were recruited if they had (1) no histories of major injury or surgery in the lower limbs and (2) no knee symptoms in the lower limbs. Healthy controls were excluded if they had musculoskeletal or neurological diseases that affect the lower limbs. A total of 27 ACLD subjects (24 males and 3 females) and 29 healthy controls (19 males and 10 females) passed the inclusion and exclusion criteria and were recruited for the study. There were no significant demographic differences between the ACLD patients and healthy controls (ACLD vs. healthy controls: age, 27.3 ± 9.1 years old vs. 25.4 ± 2.0, *p* = 0.293; gender 24 (male):3 (female) vs. 19:10, *p* = 0.058; BMI, 21.3 ± 1.6 vs. 20.6 ± 2.0, *p* = 0.169).

### Devices and experiment procedures

Kinematic knee data were collected from the participants using a three-dimensional (3D) motion capture gait system (Opti_Knee, Innomotion, Shanghai, China) (Zhang et al., 2015). The system was composed of a working station computer, a surgical navigation stereo infrared tracking device (NDI Polaris Spectra, Northern Digital, Canada), two sets of markers, a high-speed optical camera, and a handheld digitizing probe. The sampling frequency was 60 Hz. The surgical navigation stereo infrared tracking device was reported to have an accuracy level of 0.3 mm root mean square (RMS) (Elfring et al., 2010). The gait system has a repeatability of less than 1.3°in rotation and 0.9 mm in translation (Zhang et al., 2016).

**FIGURE 1:**
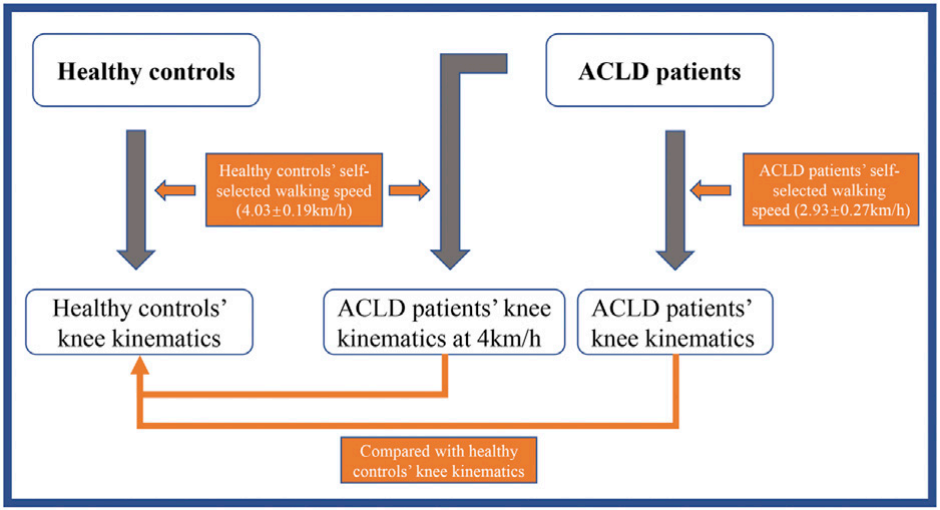
The scene of the experiment flow chart.

Before data collection, the self-selected gait speeds of the ACLD patients (ACLD speed) and healthy controls (the same speed as healthy people) were determined using the methods reported by Dingwell et al. with a slight modification (Dingwell and Marin, 2006; Nazary-Moghadam et al., 2019). Each subject walked on a level motorized bi-directional treadmill for 5 min to adapt to the treadmill. The average healthy subjects’ and ACLD and healthy subjects’ self-selected walking speeds were determined by beginning from a slow treadmill speed (i.e., 1 km/h) and slowly raising the speed until the subject stated that the speed was greater than their preferred walking speed. The treadmill speed was then decreased until the subject stated that the speed was slower than their preferred walking speed. This procedure was repeated three times. The average speeds of the three “faster” and three “slower” speed levels were calculated and discussed with each subject to determine their average preferred walking speed levels. The average self-selected walking speed of the healthy controls was 4.03 ± 0.19 km/h. The speed level of 4 km/h (nearest 4.03 ± 0.19 km/h) was selected as the healthy subjects’ walking speed level (normal speed). The average self-selected speed of ACLD subjects was 2.93 ± 0.27 km/h (ACLD speed). The flow chart is exhibited in Figure 1.

The healthy controls’ knee kinematic data were collected while they walked at their self-selected walking speeds. The ACLD patients’ knee kinematic data were collected while they walked at self-selected speeds and the speed level of healthy controls (4 km/h, normal speed), respectively. The data collecting procedure was as follows: (1) two sets of markers were fastened to the subject, and a handheld digitizing probe was used to identify bony landmarks to set up a personalized 3D coordinate system of the tibia relative to the femur with the subject in a neutral standing position (Zhang et al., 2015); (2) the healthy controls were tested with the same speed as healthy controls and the ACLD patients were tested at both the ACLD speed and the same speed as healthy peoples in random order on the treadmill; (3) before data were collected, the subjects walked on a treadmill for 1 min to ensure their gait was stable, and then kinematic data were collected while the subjects walked on the treadmill (Figure 1D) for 15 s (about 15 gait cycles, GCs); (4) the time between the tests at the two gait speeds for each ACLD subject was 5 min to avoid the influence of the previous test and fatigue effects.

The system automatically averaged the kinematics from all the gait cycles (about 15 cycles) into an averaged gait cycle. The kinematic knee data included three angular parameters and three translational parameters. The three angular parameters were adduction/abduction angle (degrees, °), flexion/extension angle (degrees, °), and internal/external rotation angle (degrees, °). The three translational parameters were anterior/posterior tibial translation (millimeters, mm), distal/proximal tibial translation (millimeters, mm), and medial/lateral tibial translation (millimeters, mm).

### Statistical analysis

One-way ANOVA was undertaken to determine which parts of gait phases of knee kinematics were significant between groups using SPSS version 22.0 (SPSS, Chicago, IL, United States). Then the averages of knee kinematics of the affected phases and range of motions (ROM) among groups were compared *via* One-way ANOVA and the posthoc tests, Dunnett-t methods (healthy controls were set as the reference group).

In a pilot study, 10 subjects (5 ACLD subjects and five healthy controls) were recruited for sample size calculation *via* PASS version 15.0 (NCSS, Kaysville, Utah, United States). We selected the range of motion (ROM) of anteroposterior translation to calculate the sample size. The average anteroposterior ROM of healthy controls was 12.3 mm. The average anteroposterior ROM of ACLD patients at the ACLD walking speed level was 16.0 mm, whereas the average anteroposterior ROM of ACLD patients while walking at the speed level of healthy people was 18.9 mm. The standard deviation of the aforementioned data was 3.5 mm. The power (1-β) requirement was set to 90%. The significance level was set to 0.05. The results showed that a sample size of at least nine patients in each group achieved 93.04% power to detect significant differences in anteroposterior translation ROM among healthy subjects, ACLD patients with ACLD speed, and ACLD patients with the speed level of healthy subjects. Therefore, the recruited number of 27 ACLD patients and 29 healthy controls met the sample size requirement for this study.

## Results

**FIGURE 2:**
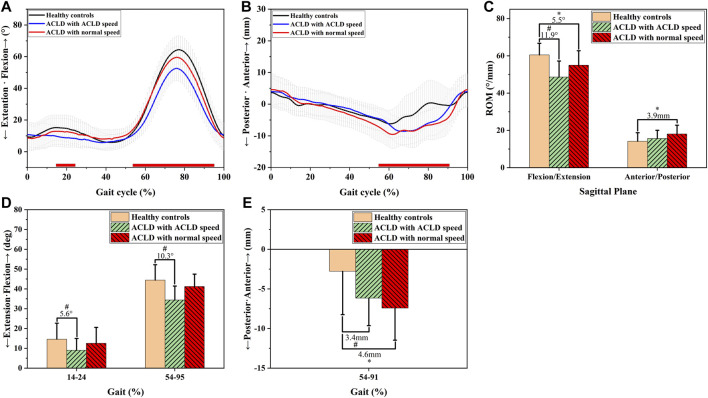
Sagittal knee kinematic alterations of healthy controls and ACLD patients during gait. Chart **(A,D)** show walking knee flexion angle alterations of ACLD patients at ACLD speed and the speed level of healthy controls (normal speed) compared to healthy controls. Chart **(B,E)** show walking anteroposterior tibial translation alterations of ACLD patients at ACLD speed and the speed level of healthy controls (normal speed) compared to healthy controls. Chart **(C)** shows the range of motion of flexion angle and anteroposterior tibial translation of ACLD patients compared to healthy controls during walking. # Significant kinematic differences were found in ACLD patients who walked at ACLD speed compared to healthy controls. * Significant kinematic differences were found in ACLD patients who walked at the same speed level as healthy controls (Normal speed) compared to healthy controls.

In the sagittal plane (Figure 2), ACLD patients at self-selected speeds had smaller knee flexion angles than healthy controls during gait (14–24% GC, 5.6°, *p* < 0.05 & 54–95% GC, 10.3°, *p* < 0.05, Figures 2A,D). In contrast, ACLD patients at normal speed (4 km/h) had no significant decreased knee flexion angle during gait (*p* > 0.05, Figures 2A,D). The ROM of flexion angle of ACLD patients at self-selected speeds was smaller than that of healthy controls (11.9°, *p* < 0.05, Figure 2C), and the ROM of flexion angle of ACLD patients at normal speed was smaller than that of healthy controls (5.5°, *p* < 0.05, Figure 2C). ACLD patients at self-selected speeds had greater posterior tibial translation than healthy controls during gait (3.4 mm, *p* < 0.05), and ACLD patients had much greater posterior tibial translation than healthy controls (4.6 mm, *p* < 0.05) during 54–91% GC (Figures 2B,E). The ROM of anteroposterior translation of ACLD patients at normal speed was greater than that of healthy controls (3.9 mm, *p* < 0.05, Figure 2C). In contrast, there was no significant difference between ACLD patients at ACLD speed and healthy controls (*p* > 0.05, Figure 2C).

**FIGURE 3:**
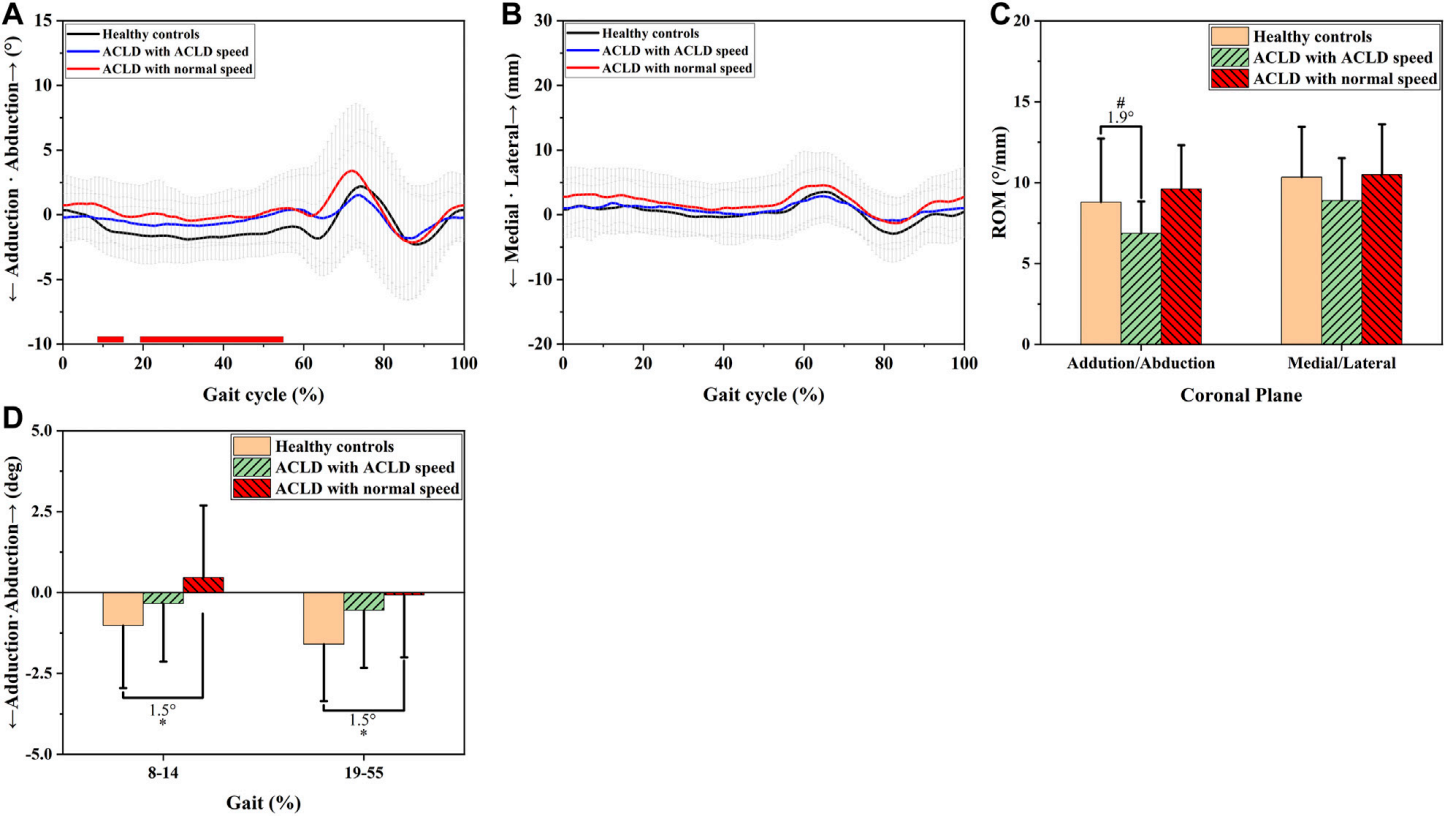
Coronal knee kinematic alterations of healthy controls and ACLD patients during gait. Chart **(A,D)** show walking adduction/abduction angle alterations of ACLD patients at ACLD speed and the speed level of healthy controls (normal speed) compared to healthy controls. Chart **(B)** shows walking medial/lateral tibial translation alterations of ACLD patients at ACLD speed and the speed level of healthy controls (normal speed) compared to healthy controls. Chart **(C)** shows the range of motion of adduction/abduction angle and medial/lateral tibial translation of ACLD patients compared to healthy controls during walking. # Significant kinematic differences were found in ACLD patients who walked at ACLD speed compared to healthy controls. * Significant kinematic differences were found in ACLD patients who walked at the same speed level as healthy controls (Normal speed) compared to healthy controls.

In the coronal plane, the ACLD patients at normal speed had greater abduction angles than the healthy controls (8–14% GC, 1.5°, *p* < 0.05 & 19–55% GC, 1.5°, *p* < 0.05, Figures 3A,D). In contrast, there was no significant difference between the ACLD patients at self-selected speeds and the healthy controls (*p* > 0.05, Figures 3A,D). The ROM of adduction angle of ACLD patients at self-selected speeds was smaller than that of healthy controls (1.9°, *p* < 0.05, Figure 3C), but there was no difference in adduction ROM between ACLD patients at normal speed and healthy controls (*p* > 0.05, Figure 3C). There was no significance of mediolateral translation among healthy controls, ACLD patients at self-selected speed and normal speed (*p* > 0.05, Figures 3B,C).

**FIGURE 4:**
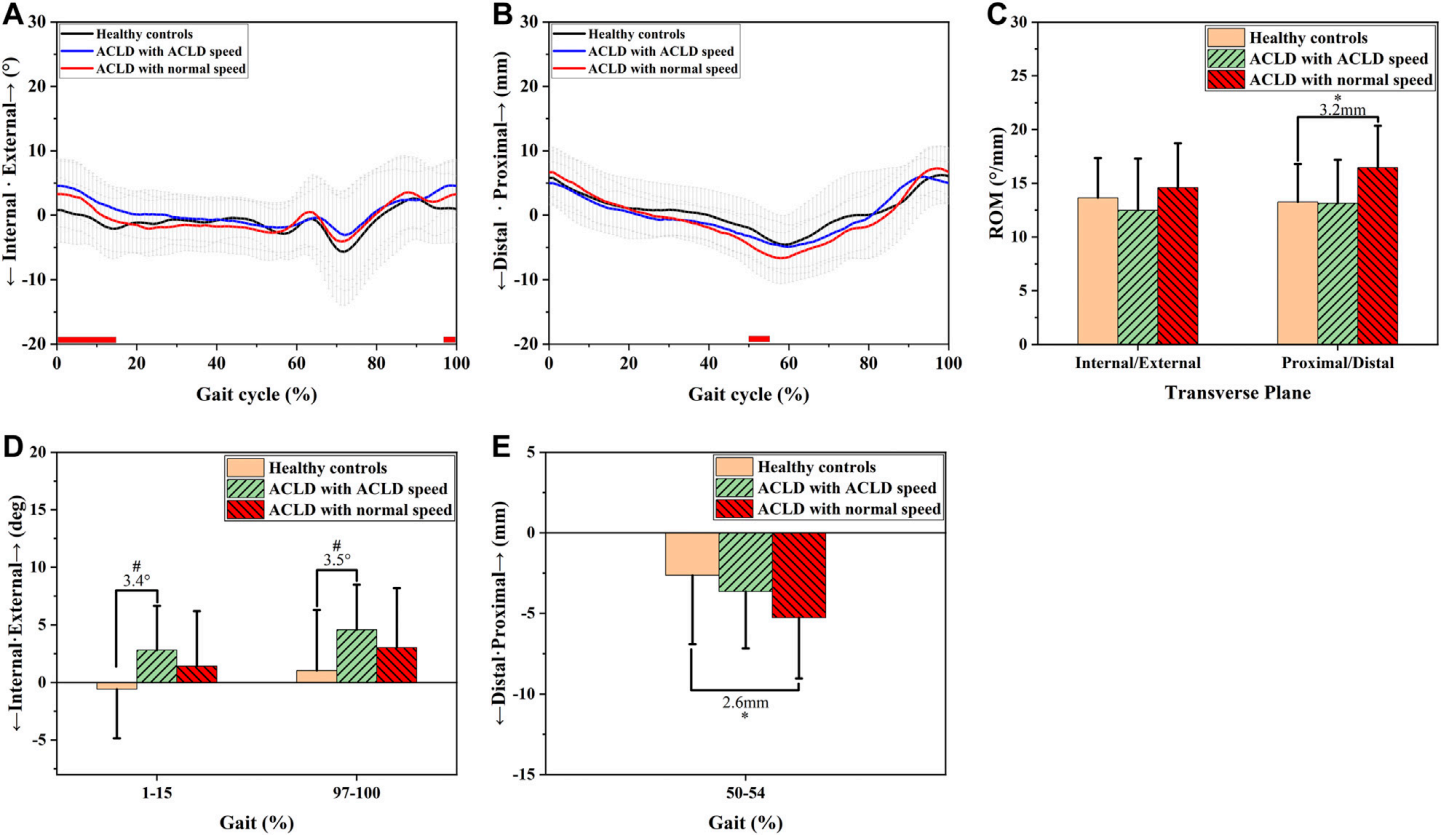
Transverse knee kinematic alterations of healthy controls and ACLD patients during gait. Chart **(A,D)** show walking internal/external tibial rotation angle alterations of ACLD patients at ACLD speed and the speed level of healthy controls (normal speed) compared to healthy controls. Chart **(B,E)** show walking distal/proximal tibial translation alterations of ACLD patients at ACLD speed and the speed level of healthy controls (normal speed) compared to healthy controls. Chart **(C)** shows the range of motion of internal/external tibial rotation angle and distal/proximal tibial translation of ACLD patients compared to healthy controls during walking. # Significant kinematic differences were found in ACLD patients who walked at ACLD speed compared to healthy controls. * Significant kinematic differences were found in ACLD patients who walked at the same speed level as healthy controls (Normal speed) compared to healthy controls.

In the transverse plane (Figure 4), the ACLD patients at self-selected speeds exhibited greater internal tibial rotation angles than the healthy controls (1–15% GC, 3.4°, *p* < 0.05 & 97–100% GC, 3.5°, *p* < 0.05, Figures 4A,D). In contrast, there was no significant difference between ACLD patients at normal speed and healthy controls (*p* > 0.05, Figures 4A,D). ACLD patients at self-selected speeds exhibited no significant difference in distal/proximal translation compared to healthy controls. In contrast, ACLD patients at normal speed had greater distal tibial translation than healthy controls (50–54% GC, 2.4mm, *p* < 0.05, Figures 4B,E). The ROM of distal/proximal translation of ACLD patients at self-selected speeds exhibited no significant difference compared to healthy controls (*p* > 0.05, Figure 4C), but ACLD patients at normal speed had greater distal/proximal translation than healthy controls (3.2 mm, *p* < 0.05, Figure 4C).

## Discussion

Non-operative treatment of ACLD has been promoted as an acceptable choice for patients without the high requirement of returning to sports (Diermeier et al., 2021). However, how to delay the progression of PTOA is an essential issue to be explored. Walking is the most frequent daily knee functional activity. Most ACLD patients are young and active people who often have busy work and fast-paced lives. As a result, they often struggle to walk faster than their self-selected comfortable speed to catch the speed of healthy subjects. Nevertheless, knee walking kinematic asymmetries have been reported to be factors in the progression of PTOA (Chaudhari et al., 2008; Andriacchi et al., 2009; Zaid et al., 2015). Walking speed significantly changes knee walking kinematics (Hanlon and Anderson, 2006; Chehab et al., 2017). Whether the walking speed of the healthy subjects leads to additional walking kinematic asymmetries remains unclear. We try to fill this gap. We aimed to explore whether the walking speed level of healthy controls leads to additional kinematic asymmetries in patients with ACLD. We hypothesized that the walking speed level of healthy subjects could increase anteroposterior knee kinematic asymmetries. Our results confirmed the hypothesis, showing that ACLD walking at the speed level as healthy controls had significant increased anteroposterior ROM (3.9 mm) compared to controls and significantly increased greater posterior tibial translation asymmetry (4.6 mm) than patients at a self-selected comfortable speed (3.4 mm) during walking (Figures 2B–D). Furthermore, our results revealed that when ACLD patients walked at the speed level of healthy controls, they had different results of abduction angles, flexion angle, tibial rotation angles, and distal tibial translation compared with patients at self-selected speed (Figures 2–4). Our findings add to the knowledge of the influence of walking speed on ACLD knees and suggest that people with ACLD knees may exhibit additional kinematic asymmetries when walking at the gait speed of healthy controls, which raises concern about health problems for patients who walk at healthy control speeds.

Consistent with previous studies (Andriacchi and Dyrby, 2005; Shin et al., 2007; Zabala et al., 2015), increased posterior tibial translation was found in ACLD patients compared to healthy controls. Furthermore, greater posterior translation and anteroposterior ROM asymmetries were exhibited in ACLD knees while walking at the speed level of healthy controls than at their self-selected speed (Figures 2B,C). The abnormal anteroposterior translation was significantly correlated with knee cartilage degeneration (Zaid et al., 2015; Kiapour et al., 2017; Ikuta et al., 2020; Li et al., 2020). Li et al. (2020) found increased anterior tibial translation in the motion of full extension to flexion 6 months after ACLR to be significantly correlated with cartilage degeneration in the medial tibia plateau at 1- and 2-year follow-ups among ACLR patients. Furthermore, Zaid et al. (2015) found the increased posterior tibial translation to have stronger effects on cartilage degenerations than anterior tibial translation in ACLR patients. The increased posterior translation exhibited by ACLD patients when walking at the same speed as healthy controls may contribute to knee cartilage degeneration. The increased anteroposterior ROM was due to the increased posterior translation during gait (Figures 2B,C). However, it was reported that the increment of anteroposterior ROM might risk the knee joint to reinjury (Gupta et al., 2020; Yamasaki et al., 2021) and affect the readiness to return to sports (Faleide et al., 2021). When walking at the speed level as healthy controls, the ACLD patients seemed to have close to “normal” (increased) knee flexion angles, especially the ROM of flexion angle (Figure 2A). However, researchers reported that increased knee flexion angles are significantly correlated with increases in quadriceps force and joint contact force, which accelerate cartilage degeneration (Besier et al., 2009; Teng et al., 2017). ACLD patients may increase their knee flexion angles as a strategy to adapt to walking at the same speed as healthy controls, but the potential effects of doing so on the knee joint should be noticed and confirmed further.

Greater adductive angles were exhibited in ACLD knees while walking at the speed level of healthy controls than at their self-selected speed (Figures 3A,D). Previous studies reported that increased abduction angle could play an important role in knee disease development. Crema et al. (2012) found that abduction or adduction malalignment (< 178° or > 182°) could be related to meniscus extrusion and, thus, increased rates of meniscus injury. Sharma et al. (2001) found that increased abduction angle could be associated with increased odds of osteoarthritis development. Therefore, the increased abduction angle at the speed level of healthy subjects could potentially hurt the knee joint of ACLD patients.

In the transverse plane, greater distal tibial translation asymmetries were exhibited in ACLD knees at the same speed as healthy controls than ACLD speed (Figures 4B,C). This could be related to the increment of posterior tibial translation asymmetry in ACLD knees at healthy walking speed compared to that at ACLD walking speed (Figure 2B). There is a natural tibial posterior slope in the knee structure (Cooper et al., 2019). Hence, once the tibia moved posteriorly, the center of femoral condyles rose relatively; thus, distal tibial translation increased. However, this is only speculation based on the anatomy of the knee joint. Whether this mechanism exists needs further exploration. The internal tibial rotation of ACLD patients was relatively restored when they walked at the speed level of healthy controls rather than the ACLD speed. Consistent with our study, Chehab et al. (2017) reported tibial internal rotation angle increased as the gait speed increased during the stance phase. This could be related to increased tibial internal moments along with the increased gait speed during the stance phase (Chehab et al., 2017).

Gait speed affects knee kinematics significantly (Lelas et al., 2003; Bejek et al., 2006; Hanlon and Anderson, 2006; Chehab et al., 2017). The present study further found that patients with ACLD at the speed level of healthy subjects had greater knee kinematic asymmetries than patients at self-selected speed, including, greater posterior tibial translation, distal tibial translation, and abduction angle asymmetries (Figures 2–4). These findings could be meaningful for ACLD patients accepting non-operative treatments and keeping their knee joints as possible as healthy and away from knee kinematics asymmetries during their daily lives. The present study has some limitations that should be noticed. Firstly, the recruited number of males was greater than females in the present study. This may bring gender bias to the study. However, the recruited gender characteristics are consistent with the gender differences in the prevalence of ACLD (Lyman et al., 2009; Ellison et al., 2021). Secondly, this study didn’t explore the long-term effects of healthy subjects’ speed levels on ACLD patients’ knee kinematics and health. The effects of these kinematic asymmetries should be investigated further in future studies.

## Conclusion

Our study showed that ACLD patients exhibited more knee kinematic asymmetries when they walked at the gait speed of healthy controls than at self-selected speeds. The kinematic asymmetries included greater posterior tibial translation (up to 4.6 mm), distal tibial translation (up to 3.2 mm), and abduction angle (up to 1.5°). These findings are meaningful for developing non-operative treatment strategies for patients with ACLD. To get fewer knee kinematic asymmetries, self-selected walking speed could be suggested for patients with ACLD during their daily lives rather than the speed levels of healthy subjects.

## Data Availability

The data are available from the corresponding authors upon a reasonable request.
